# 2000年与2010年四川大学华西医院肺癌临床诊治特征的对比研究

**DOI:** 10.3779/j.issn.1009-3419.2012.06.06

**Published:** 2012-06-20

**Authors:** 晓军 姚, 伦旭 刘, 洪伟 张, 强 蒲, 虎 廖

**Affiliations:** 1 610041 成都，四川大学华西医院胸外科 Department of Thoracic Surgery, West China Hospital, Sichuan University, Chengdu 610041, China; 2 620010 眉山，四川省眉山肿瘤医院胸外科 Department of Thoracic Surgery, Meishan Cancer Hospital, Meishan 620010, China

**Keywords:** 肺肿瘤, 病理学, 诊断, 治疗, Lung neoplasms, Pathology, Diagnosis, Therapeutics

## Abstract

**背景与目的:**

原发性支气管肺癌是最常见的恶性肿瘤之一，本研究旨在回顾性分析2000年与2010年四川大学华西医院原发性支气管肺癌临床特征的变化，为肺癌的早期诊断和治疗提供参考。

**方法:**

收集2000年与2010年在四川大学华西医院住院的四川地区常住人口中初诊原发性支气管肺癌病例，对两组患者的主要就诊原因、发病到就诊时间、伴随基础疾病、合并肺癌的双原发癌、家族恶性肿瘤史、肿瘤位置、分化程度、肿瘤分期及首诊主要治疗方式等临床资料进行对比分析。

**结果:**

收集有细胞学或组织学依据的肺癌病例共2, 167例，其中2000年616例，2010年1, 551例。10年中因体检而就诊的肺癌患者构成比上升（5.2% *vs* 16.7%, *P* < 0.001），肺癌患者发病到就诊时间缩短（*P* < 0.001），伴家族恶性肿瘤史的肺癌患者增多（3.9% *vs* 13.7%, *P* < 0.001）；鳞癌低分化癌构成比明显增加（59.4% *vs* 76.7%, *P*=0.002），而腺癌低分化癌构成比明显减少（72.3% *vs* 51.8%, *P*=0.002）；非小细胞肺癌患者Ⅰa期及Ⅳ期构成比明显上升（Ⅰa期：1.0% *vs* 4.5%, *P* < 0.001; Ⅳ期：30.4% *vs* 37.8%, *P* < 0.001），Ⅲa期构成比明显下降（26.6% *vs* 14.8%, *P*=0.002）；治疗上非小细胞肺癌患者首诊采取化疗构成比上升（35.9% *vs* 42.4%, *P*=0.007），Ⅲa期首诊采取手术者明显上升（41.8% *vs* 63.4%, *P*=0.002），Ⅳ期首诊采取手术者明显下降（9.4% *vs* 3.1%, *P*=0.001），小细胞肺癌患者首诊采取手术者明显下降（30.4% *vs* 4.3%, *P* < 0.001）。

**结论:**

近十年肺癌患者的临床特征部分已产生了较为明显的变化，根据这些变化选择更适合的预防、诊断和治疗措施对降低肺癌发病率、提高生存率有一定意义。

## Abstract (en)

**Background and objective:**

Primary lung cancer is one of the most common malignant tumors. The aim of the current study is to retrospectively analyze the clinical features variation of patients with primary bronchogenic carcinoma in West China Hospital Sichuan University to provide information for early detection and treatment of lung cancer.

**Methods:**

We collected data of patients of permanent population in Sichuan province who diagnosed primary bronchogenic carcinoma in 2000 and 2010 in West China Hospital Sichuan University respectively for comparative analysis of reasons to visit the doctor, duration from symptom onset to visit the doctor, combined diseases, incidences of bi-primary carcinoma, family history of malignant tumor, sites of tumors, grade of differentiation, tumor staging and initial treatment modalities.

**Results:**

A total of 2, 167 cases (616 cases in 2000 and 1, 551 cases in 2010) met inclusion criteria were retrieved for analysis. In 2010, compared with data of 2000, the rate of patients who visit the doctors because abnormalities were detected by health examination elevated remarkably (5.2% *vs* 16.7%, *P* < 0.001), the duration from symptom onset to visit the doctor abridged significantly (*P* < 0.001), patients with family history of malignant tumor increased significantly (3.9% *vs* 13.7%, *P* < 0.001), the constituent ratio of poorly differentiated adenocarcinoma decreased (72.3% *vs* 51.8%, *P*=0.002) accompanied with low differentiated squamous cell carcinoma increased (59.4% *vs* 76.7%, *P*=0.002). For NSCLC staging, there is a notably increase of rate of stage Ⅰa (1.0% *vs* 4.5%, *P* < 0.001) and stage Ⅳ (30.4% *vs* 37.8%, *P* < 0.001) while decrease of stage Ⅲa (26.6% *vs* 14.8%, *P*=0.002). For initial treatment modalities, there is markedly increased chemotherapy rate of non-small cell lung cancer (NSCLC) patients (41.8% *vs* 63.4%, *P*=0.002) while remarkably increased surgery rate of stage Ⅲa patients (41.8% *vs* 63.4%, *P*=0.002) and decreased surgery rate of stage Ⅳ patients (9.4% *vs* 3.1%, *P*=0.001). The surgery rate of small cell lung cancer (SCLC) patients decrease sharply (30.4% *vs* 4.3%, *P* < 0.001).

**Conclusion:**

There clinical features of lung cancer patients were significantly changed in the past ten years, new prevention, diagnosis and treatment strategies are needed to accommodate the variation.

肺癌目前是全球发病率和死亡率最高的恶性肿瘤，2008年全球新发病例预测约161万例，死亡约138万例，分别占恶性肿瘤新发病例及死亡病例的13%及18%^[1]^。我国肺癌的发病率及死亡率已居所有恶性肿瘤之首^[2]^。尽管目前的诊治水平及医疗设施较前有了极大的改进，但由于肺癌的解剖特殊性，约80%的肺癌患者就诊时已属晚期，失去了手术机会，5年生存率仅维持在15%左右^[3]^。若对早期肺癌患者施行根治性手术，其5年生存率可达80%以上^[4]^。所以，肺癌的早期诊断和治疗显得尤为重要。本文将对2000年与2010年在四川大学华西医院住院的四川地区常住人口中2, 167例初诊原发性支气管肺癌进行比较研究，以初步了解近十年来肺癌临床特征的变化，为肺癌的早期诊断和治疗提供参考。

## 1 资料和方法

### 1.1 病例资料

来源于四川大学华西医院信息科登记的住院肺恶性肿瘤病例。纳入标准：2000年与2010年在四川大学华西医院住院的四川地区常住人口中初诊肺恶性肿瘤病例。排除标准：①非四川地区常住人口；②肺转移性恶性肿瘤；③临床诊断肺恶性肿瘤，但无确切病理学依据；④复治及复发病例。共收集有明确病理依据的肺癌病例2, 167例，其中2000年616例，2010年1, 551例。细胞或组织学依据来源于痰液、胸水、纤支镜检、转移淋巴结或肿瘤活检、肺穿刺活检、纵隔镜及胸腔镜活检、手术切除标本等。病理类型均按WHO肺部肿瘤组织学分类1999年版标准进行分类录入。肺癌分期均按1997版肺癌国际分期标准录入，包括临床分期（cTNM-cStage）及病理分期（pTNM-pStage）。收集内容包括两组患者的主要就诊原因、发病到就诊时间、伴随基础疾病、合并肺癌的双原发癌、家族恶性肿瘤史、肿瘤位置、分化程度、肿瘤分期及首诊主要治疗方式等临床资料。

### 1.2 统计分析方法

利用Excel软件建立数据库并录入所有资料，数据经校对无误后导入SPSS 16.0软件进行统计分析。采用*χ*^2^检验、秩和检验进行组间差异比较。*P* < 0.05为差异有统计学意义。

## 2 结果

### 2.1 主要就诊原因

2000年与2010年肺癌患者就诊时主要首发症状所占比重分别为：咳嗽（72.6% *vs* 59.3%）、咯血（35.2% *vs* 21.5%）、胸背痛（24.8% *vs* 19.0%）、胸闷气紧（18.0% *vs* 14.8%）、声嘶（3.4% *vs* 2.5%）、发热（2.9% *vs* 1.8%）、四肢关节痛（2.8% *vs* 2.7%）、呼吸困难（1.1% *vs* 0.3 %）、其它（2.9% *vs* 4.5%）。10年间因体检而就诊的肺癌患者构成比明显提高（5.2% *vs* 16.7%, *χ*^2^=50.192, *P* < 0.001）。

### 2.2 发病到就诊时间

2000年肺癌患者发病到就诊中位时间为3个月（3天-60个月），2010年肺癌患者发病到就诊中位时间为2个月（1天-60个月），较2000年明显缩短（*P* < 0.001）。10年间发病到就诊的时间在1月内的肺癌患者构成比明显升高（11.7% *vs* 19.4%, *χ*^2^=18.433, *P* < 0.001）。

### 2.3 伴随基础疾病

2000年与2010年肺癌患者伴随的相关基础疾病所占比重分别为慢性阻塞性肺疾病（12.6% *vs* 16.6%, *χ*^2^=5.213, *P*=0.022）、肺部感染（7.5% *vs* 14.1%, *χ*^2^=17.866, *P* < 0.001）、肺结核（2.9% *vs* 0.7%, *χ*^2^=16.351, *P* < 0.001）、高血压（7.5% *vs* 11.7%, *χ*^2^=8.526, *P*=0.004）、糖尿病（4.9% *vs* 8.2%, *χ*^2^=7.223, *P*=0.007）、冠心病（1.5% *vs* 2.1%, *χ*^2^=1.031, *P*=0.310）、心律失常（0.3% *vs* 1.5%, *χ*^2^=5.187, *P*=0.023）。其中，冠心病所占比重的差异无统计学意义（*P*>0.05），2010年慢性阻塞性肺疾病、肺部感染、高血压、糖尿病、心律失常所占比重上升，肺结核所占比重下降。

### 2.4 合并肺癌的双原发癌

2000年有7例（1.1%）肺癌患者诊断双原发癌，其中1例同期发病（肾癌），6例异时发病，按两次疾病诊断间隔时间罗列依次为：食管癌（2年）、喉癌（3年）、甲状腺癌（3年）、鼻咽癌（3年）、乳腺癌（5年）、结肠癌（6年）各1例，有4例施行肺癌手术。2010年有29例（1.9%）肺癌患者诊断双原发癌，其中2例同期发病（腮腺癌及恶性黑色素瘤各1例），27例异时发病，按两次疾病诊断间隔时间罗列依次为：宫颈癌3例（3个月、2年、2年）、腮腺癌（8个月）、子宫原位癌（2年）、鼻咽癌3例（2年、2年、6年）、胆管细胞癌（3年）、胃癌（3年）、肺癌3例（3年、6年、12年）、乳腺癌2例（3年、18年）、喉癌2例（4年、4年）、肝癌（5年）、结肠癌2例（5年、5年）、直肠癌4例（6年、6年、7年、12年）、肾癌2例（7年、10年）、子宫内膜癌(43年），有14例施行肺癌手术。两个时间段诊断双原发癌的肺癌患者构成比的差异无统计学意义（*χ*^2^=1.452, *P*=0.228）。

### 2.5 家族恶性肿瘤史

2000年与2010年分别有24例（3.9%）及212例（13.7%）肺癌患者有一级亲属的恶性肿瘤史，其构成比明显增加（*χ*^2^=43.388, *P* < 0.001）。在这些家族恶性肿瘤史中，两个时间段均以消化道肿瘤（41.7% *vs* 42.0%）及肺癌（25.0% *vs* 35.8%）最多见。

### 2.6 肿瘤位置分布

如表 1所示，2000年与2010年肺癌在不同肺叶的频数分布变化无统计学差异。除去不能判断原发肺叶的双肺病变，2000年左右肺叶构成比分别为45.9%和53.6%，2010年分别为45.0%和54.1%，差异无统计学意义（*χ*^2^=0.100, *P*=0.752）。2000年上叶及中下叶构成比分别为51.8%和46.1%，2010年分别为52.5%和45.2%，差异无统计学意义（*χ*^2^=0.128, *P*=0.721）。两个时间段肺癌的位置分布特点均为右肺高于左肺，上叶高于中下叶。

**1:** 肺癌在不同肺叶的频数分布 Frequencies of lobe distribution of lung cancer in different times

Lobe distribution	2000 (*n*=616)	2010 (*n*=1, 551)	*χ*^2^	*P*
LUL	161	416	0.106	0.745
LLL	118	269	0.987	0.320
RUL	158	399	0.001	0.971
RLL	118	315	0.367	0.545
RML	48	117	0.039	0.844
LMB	4	13	0.032	0.858
RMB	6	8	1.442	0.230
Both	3	14	0.517	0.472
LUL: left upper lobe; LLL: left lower lobe; RUL: right upper lobe; RLL: right lower lobe; RML: right middle lobe; LMB: left main bronchus; RMB: right main bronchus.				

### 2.7 肿瘤分化程度

2000年与2010年在非小细胞肺癌（non-small cell lung cancer, NSCLC）中分别有186例（29.4%）及464例（29.9%）病理诊断标明分化程度。如表 2所示，2000年与2010年不同病理分化程度所占比重由高到低依次均为：高分化（2.2% *vs* 0.6%）、中分化（32.3% *vs* 36.4%）、低分化（65.6% *vs* 62.9%），各分化程度构成比无统计学差异（*χ*^2^=1.56, *P*=0.212; *χ*^2^=0.820, *P*=0.365; *χ*^2^=0.291, *P*=0.590）。腺癌中分化癌构成比由27.7%升至47.5%（*χ*^2^=8.724, *P*=0.003），低分化癌构成比由72.3%下降至51.8%（*χ*^2^=9.379, *P*=0.002）。鳞癌中分化癌构成比由35.8%降至23.3%（*χ*^2^=6.412, *P*=0.011），低分化癌构成比由59.4%升至76.7%（*χ*^2^=10.049, *P*=0.002）。各病理类型中高分化癌数量极少，不做对比。

**2:** 肺癌病理类型分化程度的频数分布 Frequencies of tumor differentiation in different histological types of lung cancer

Differentiation	2000					2010			
	AD	SCC	Else	Total		AD	SCC	Else	Total
High	0	4	0	4		2	0	1	3
Medium	18	39	3	60		122	44	3	169
Low	47	63	12	122		133	145	14	292
AD : adenocarcinoma; SCC : squamous cell carcinoma.									

### 2.8 肿瘤分期

如表 3所示，在NSCLC中2000年Ⅰa期占1.0%，Ⅰb期占14.7%，Ⅱa期占1.0%，Ⅱb期占5.6%，Ⅲa期占26.6%，Ⅲb期占20.7%，Ⅳ期占30.4%；2010年Ⅰa期占4.5%，Ⅰb期占15.5%，Ⅱa期占1.3%，Ⅱb期占7.0%，Ⅲa期占14.8%，Ⅲb期占19.1%，Ⅳ期占37.8%。2010年与2000年相比，Ⅰa期及Ⅳ期患者构成比明显上升（*χ*^2^=15.351, *P* < 0.001; *χ*^2^=38.753, *P* < 0.001），Ⅲa期患者构成比明显下降（*χ*^2^=9.940, *P*=0.002），其余分期患者构成比差异无统计学意义（Ⅰb期：*χ*^2^=0.230, *P*=0.631；Ⅱa期：*χ*^2^=0.320, *P*=0.572；Ⅱb期：*χ*^2^=1.308, *P*=0.253；Ⅲb期：*χ*^2^=0.701, *P*=0.402）；Ⅲ期-Ⅳ期患者构成比在两个时间段分别为77.7%及71.7%，下降明显（*χ*^2^=7.826, *P*=0.005）。在小细胞肺癌中广泛期患者构成比在两个时间段分别为43.5%及57.3%，差异无统计学意义（*χ*^2^=1.584, *P*=0.208）。

**3:** 肺癌分期与首诊治疗方式的频数分布关系 Relationship between the staging of lung cancer and the treatment modality

Stage	2000							2010					
	Surgery	Inter-therapy	Radio- therapy	Chemo-therapy	Else	Total		Surgery	Inter- therapy	Radio- therapy	Chemo- therapy	Else	Total
NSCLC													
Ⅰa	6	0	0	0	0	6		55	0	0	4	3	62
Ⅰb	75	1	1	6	4	87		176	0	2	18	16	212
Ⅱa	5	1	0	0	0	6		16	0	0	1	1	18
Ⅱb	30	0	0	2	1	33		84	0	1	7	3	95
Ⅲa	66	21	4	53	14	158		128	3	6	74	7	202
Ⅲb	18	13	7	60	25	123		53	2	24	140	42	261
Ⅳ	17	18	15	92	38	180		16	5	29	340	126	516
Total	217	54	27	213	82	593		528	10	62	564	198	1, 366
SCLC													
Limited	6	2	1	3	1	13		7	0	8	51	13	79
Extensive	1	0	1	4	4	10		1	1	10	75	19	106
Total	7	2	2	7	5	23		8	1	18	126	32	185
NSCLC: non-small cell lung cancer; SCLC: small cell lung cancer.													

### 2.9 首诊主要治疗方式

如表 3所示，在NSCLC患者中2000年与2010年首诊采取手术的患者构成比分别为36.6%及38.7%，差异无统计学意义（*χ*^2^=0.744, *P*=0.388），而化疗患者构成比由35.9%升至42.4%（*χ*^2^=7.181, *P*=0.007），介入治疗（包括支气管动脉栓塞及支气管动脉造影化疗）患者构成比由9.1%降至0.7%（*χ*^2^=91.756, *P* < 0.001），放疗及其它治疗的患者构成比差异无统计学意义（*χ*^2^=0.000, *P*=0.989; *χ*^2^=0.160, *P*=0.689）。2000年与2010年不同肿瘤分期中首诊采取手术的患者构成比对比如下：Ⅰ期无明显变化（87.1% *vs* 84.3%, *χ*^2^=0.424, *P*=0.515），Ⅱ期无明显变化（89.7% *vs* 88.5%, *χ*^2^=0.045, *P*=0.831），Ⅲa期明显上升（41.8% *vs* 63.4%, *χ*^2^=9.808, *P*=0.002），Ⅲb期无明显变化（14.6% *vs* 20.3%, *χ*^2^=1.785, *P*=0.182），Ⅳ期明显下降（9.4% *vs* 3.1%, *χ*^2^=11.890, *P*=0.001）。小细胞肺癌患者中首诊采取手术的患者构成比由30.4%降至4.3%（*χ*^2^=20.842, *P* < 0.001），而化疗患者构成比由30.4%上升至68.1%（*χ*^2^=7.181, *P*=0.007）。

## 3 讨论

肺癌首发症状以咳嗽、咯血、胸背痛及胸闷气紧等局部症状多见，其中以咳嗽最为常见，本组资料亦如此。相较于2000年，2010年因体检而就诊的肺癌患者构成比明显升高，发病到就诊时间在1个月内的肺癌患者构成比也明显升高，通常这部分肺癌患者分期较早，预后也较好。肺癌症状无特异性，通常症状由轻到重，由不典型到典型。而且，肺癌高发于老年患者，我国老年群体普遍健康意识较差，多数肺癌患者在出现咳嗽、咯血等症状后均未至正规医院就诊，至确诊时约80%的患者已为晚期，导致病情延误，丧失手术时机。为提高肺癌患者的早期诊断及治疗应加大对肺癌的科普宣传，提高人们对肺癌的认识尤其是对早期症状的认识，从而缩短患者就诊和确诊的时间，提高患者的生存质量，并延长患者的生存时间。

多数肺癌患者就诊时伴有多器官或系统疾病尤其是老年患者。研究^[5-7]^显示初诊肺癌患者约40%-70%合并慢性阻塞性肺疾病，且近年来增加趋势明显，本资料数据远低于此，其数值的差异可能与慢性阻塞性肺疾病的诊断标准差异有关。肺癌患者伴随重要器官或系统的基础疾病与治疗及预后密切相关，合并基础疾病的患者术后并发症发生几率是不合并基础疾病患者的8倍左右^[8]^。本组资料十年间相关基础疾病如慢性阻塞性肺疾病、肺部感染、高血压、糖尿病、心律失常等在肺癌患者中所占比重明显上升，提醒我们应更加注重多学科协同治疗，这对减少因基础疾病导致的并发症并提高肺癌患者生存质量具有重要的临床意义。

多原发癌是指人体不同或同一器官出现两个或两个以上独立的肿瘤病灶，其病理类型相同或不同。本资料多原发癌依据Warren^[9]^制定的诊断标准，多原发肺癌依据2003年美国胸科医师协会推荐的诊断标准^[10]^。文献报道多为双原发癌，两个以上者极为少见。本资料10年间合并肺癌的双原发癌构成比虽有升高趋势，但无统计学差异。流行病学研究^[11]^显示多原发癌的发生可能会增加，其原因可能与患者肿瘤易感性、第一原发癌历经放化疗后免疫功能下降、患者平均寿命的延长及肿瘤诊断水平的提高等多种因素有关。在临床中即使多原发癌的诊断标准为多数人所采用，但尚存争议，也常会出现难以鉴别到底是原发肿瘤还是转移瘤的情况，尤其是在间隔时间较短、病理类型相同的情况下区分多肺部肿瘤为原发还是转移，目前尚无统一标准。因此，对于同时发现的两个肺癌病灶及第一原发癌后短期出现的肺内新发病灶不能一概认为就是肺内转移，在不能获取病理依据的情况下必要时可行胸腔镜活检或开胸探查，并根据术中冰冻病理决定具体术式。如果术前都将其归为晚期患者，部分患者必将贻误最佳手术时机。

有恶性肿瘤家族史的人群发生恶性肿瘤的危险性明显高于普通人群，即肿瘤的家族聚集性。多项研究^[12-15]^显示，家族恶性肿瘤史与肺癌的发生发展密切相关。在过去由于经济水平、医疗条件的限制及人群健康意识的普遍欠缺，肺癌患者就诊时所提供的家族史（尤其是父母恶性肿瘤史）缺乏完整性及真实性。而目前鉴于上述情况的改善，恶性肿瘤的检出率已不断提高。同时，各种致病因素的综合作用也导致了近年来恶性肿瘤（尤其是肺癌）的发病率上升。以上诸多因素可能导致了本资料近10年伴有一级亲属恶性肿瘤史的肺癌患者构成比增加。因此，对有恶性肿瘤家族史者的人群应加大宣传及筛查力度，使该部分人群定期体检，在罹患肺癌等恶性肿瘤时能及早治疗。

肺癌发病部位以右肺高于左肺、上叶高于中下叶，本组资料10年间肺癌在各肺叶的分布情况无明显变化。由于近年来肺癌发病年龄有年轻化的趋势，且肺结核也有发病率升高的迹象^[16]^，中青年患者上叶尖后段及下叶背段的病灶常被误认为是肺结核，在缺乏足够结核证据的情况下，应尽量获取病理标本以排除肺癌，必要时行剖胸探查组织活检，以免延误病情。而即使诊断为肺结核，在正规抗结核治疗过程中，病灶持续增大或出现纵隔淋巴结肿大及新发病灶，应高度警惕结核和肺癌共存，因其在临床中并不少见。

肺癌的分化程度多用于鳞癌和腺癌，在鳞癌和腺癌中均以低分化癌最为多见。鳞癌多见于男性及吸烟患者，生长缓慢，对放化疗相对较敏感，在各病理类型中预后较好；而腺癌多见于女性及非吸烟患者，生长较慢，对放疗不敏感，较早发生血行转移，预后较差^[17]^。本资料10年间鳞癌的低分化癌构成比明显增加，而腺癌的低分化癌构成比明显减少，是否预示着鳞癌的恶性程度在增加，反之，腺癌的恶性程度在降低？这种变化对鳞癌和腺癌患者的远期生存率是否会产生一定的影响？那么，又是何种因素造成了这种变化，烟草亦或环境？因此，对于鳞癌和腺癌分化程度的变化尚需多中心、大样本资料来印证，两者之间的预后差异值得重新审视。

肺癌患者起病隐匿，至确诊时多数已为晚期，本组资料Ⅲ期、Ⅳ期患者构成比两个时间段均在70%以上，但其构成比近10年明显下降，而Ⅰa期患者构成比明显升高，其中半数以上（56.5%）由体检发现，其变化可能与资料中肺癌患者发病到就诊时间的缩短及体检患者构成比的增加有关。在临床中，很多所谓的早期NSCLC术后很快发生转移，证明其分期偏早，其实术前可能已存在远处转移，只是未检测出来而已，应定为Ⅳ期。目前，由于检测手段的丰富尤其是正电子发射体层扫描（positron emission tomography-computed tomography, PET-CT）可发现胸外转移灶，或对侧肺门、纵隔及锁骨上的N3淋巴结转移，使临床定期更为准确，由此可能导致了资料中近10年Ⅳ期及小细胞肺癌（small cell lung cancer, SCLC）中广泛期肺癌患者构成比的升高。因此，应对肺癌患者进行周密的检测，如CT、MRI、骨扫描、PET-CT、纵隔镜、超声支气管镜（endobronchial ultrasound-guided transbronchial needle aspiration, EBUS-TBNA）及食管镜（endoscopic ultrasound-guided fine needle aspiration, EUS-FNA）等，以正确地进行分期，严格掌握手术适应症，使患者从中受益。

肺癌治疗首选外科手术，根治性手术至今仍是唯一有可能使肺癌患者获得治愈的治疗方式。在NSCLC中手术适应于临床分期为Ⅰ期、Ⅱ期及Ⅲa期的病例，但对于部分Ⅲb期及Ⅳ期（孤立性脑、肾上腺及肺内转移）病例，也可施行姑息性手术或以手术为主的综合治疗。本组资料10年间Ⅲa期肺癌患者中首诊采取手术的患者构成比明显上升，而Ⅳ期明显下降，显示该院对于局部晚期肺癌（T3、N2）的手术趋于积极，对于有远处转移的Ⅳ期患者手术则更为慎重。而对于可切除的侵犯纵隔重要结构（心包、心脏、大血管、食管和隆凸）的T4及N3分期的NSCLC毕竟是少数，其中一部分可通过新辅助化疗来降低T、N分期，以提高切除率及远期生存率^[18]^。对于SCLC的手术选择则要求更严，其术后也一律辅助化疗。一项基于SEER数据的研究结果^[19]^显示，15, 384例小细胞肺癌患者中有1, 469例（9.5%）接受了手术治疗，这些接受手术治疗患者的中位生存期明显优于未接受手术患者。但也有研究^[20]^显示，对于小细胞肺癌无论是局限期还是广泛期，手术均不能明显改善患者的生存期。总之，手术治疗在小细胞肺癌治疗中的价值仍未达成共识，而本组资料小细胞肺癌患者首诊采取手术者构成比近10年已大幅下降。肺癌化疗的临床研究较为活跃，国内外文献近期出现了较多的联合方案，肺癌化疗取得了可喜的进展，新辅助化疗也是近年来的一个研究热点，化疗在肺癌综合治疗中的重要地位日渐提升，这种变化在本资料中也得到了体现。经血管介入治疗（包括介入化疗和介入栓塞）主要应用于中晚期肺癌患者，是一种姑息性局部治疗，疗效有限，远期效果均不理想，且不能替代手术、放疗和化疗。因此对于肺癌患者，介入治疗仅能作为综合治疗方法之一。本组资料NSCLC患者首诊采取经血管介入治疗者构成比近10年明显下降，其原因可能与介入治疗的局限性及近年来外科手术和放化疗技术的进展有关。

近10年肺癌患者的临床特征部分已产生了较为明显的变化，部分则不明显，根据这些变化选择更适合的预防、诊断和治疗措施对降低肺癌发病率、提高生存率有一定意义。
